# Tristhiolato Pseudopeptides Bind Arsenic(III) in an AsS_3_ Coordination Environment Imitating Metalloid Binding Sites in Proteins

**DOI:** 10.1021/acs.inorgchem.3c00563

**Published:** 2023-04-18

**Authors:** Levente I. Szekeres, Pascale Maldivi, Colette Lebrun, Christelle Gateau, Edit Mesterházy, Pascale Delangle, Attila Jancsó

**Affiliations:** † Department of Inorganic and Analytical Chemistry, University of Szeged, Dóm tér 7, Szeged H-6720, Hungary; ‡ CEA, CNRS, Grenoble INP, IRIG, SyMMES, Universite Grenoble Alpes, Grenoble 38000, France

## Abstract

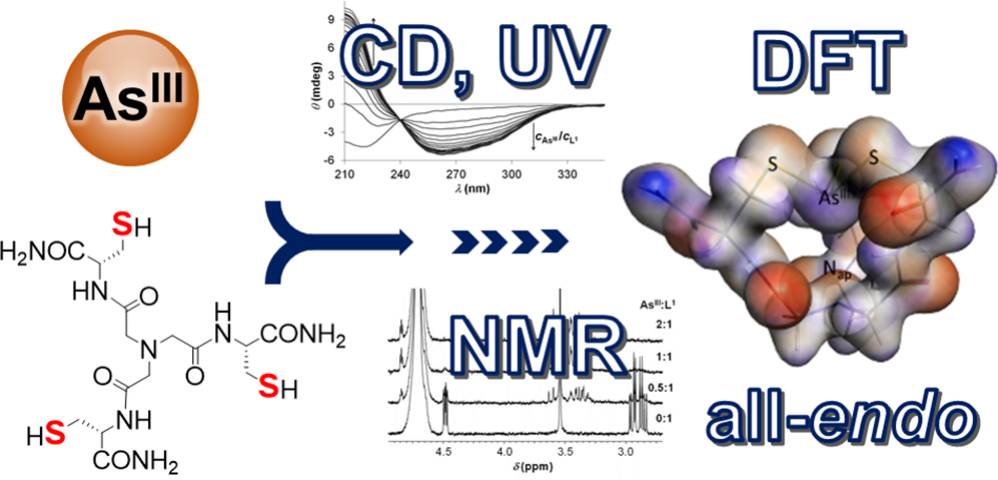

The As^III^ binding of two NTA-based tripodal pseudopeptides, possessing three cysteine (ligand **L^1^**) or d-penicillamine residues (ligand **L^2^**) as potential coordinating groups for soft semimetals or metal ions, was studied by experimental (UV, CD, NMR, and ESI-MS) and theoretical (DFT) methods. All of the experimental data, obtained with the variation of the As^III^:ligand concentration ratios or pH values in some instances, evidence the exclusive formation of species with an AsS_3_-type coordination mode. The UV-monitored titration of the ligands with arsenous acid at pH = 7.0 provided an absorbance data set that allowed for the determination of apparent stability constants of the forming species. The obtained stabilities (log*K*′ = 5.26 (As**L^1^**) and log*K*′ = 3.04 (As**L^2^**)) reflect high affinities, especially for the sterically less restricted cysteine derivative. DFT calculated structures correlate well with the spectroscopic results and, in line with the ^1^H NMR data, indicate a preference for the all-*endo* conformers resembling the As^III^ environment at the semimetal binding sites in various metalloproteins.

## Abstract 2

Tripodal ligands bearing three Cys or d-Pen arms exclusively stabilize species with an AsS_3_ coordination. Spectroscopic and theoretical results indicate a preference for the all-*endo* conformer resembling the As^III^ coordination environment in the three cysteine-bound As^III^ semimetal binding sites in metalloproteins.

## Introduction

Tristhiolato coordination sites are very important in metalloregulation or detoxification in biology, for instance, for soft metal ions, such as Hg^II^ in the regulatory proteins MerR^1^ or Cu^I^ when trapped in metallothioneins.^2^ Interestingly, MS_3_ sites are also widely found in arsenic-binding proteins.^3−6^ For As^III^, a metalloid with a lone electron pair, a wide range of interactions may dictate the structure of the coordination site. Indeed, the As^III^S_3_ center may be in an *endo*- or *exo*-type coordination,^7^*endo* and *exo* referring to the relative orientation of the carbon atoms of the alpha methylene groups and As^III^ as compared to the S_3_ plane (same side, *endo*; opposite side, *exo*). Repulsive forces between the sulfur lone pairs,^7^ steric hindrance due to close proximity of bulky hydrophobic groups around the As^III^ lone pair,^7^ and secondary bonding interactions between As^III^ and various heteroatoms^8−10^ or π-systems^9−13^ or σ-hole bonding^14,15^ may all affect the stability and structure of As^III^ complexes.

**Scheme 1:**
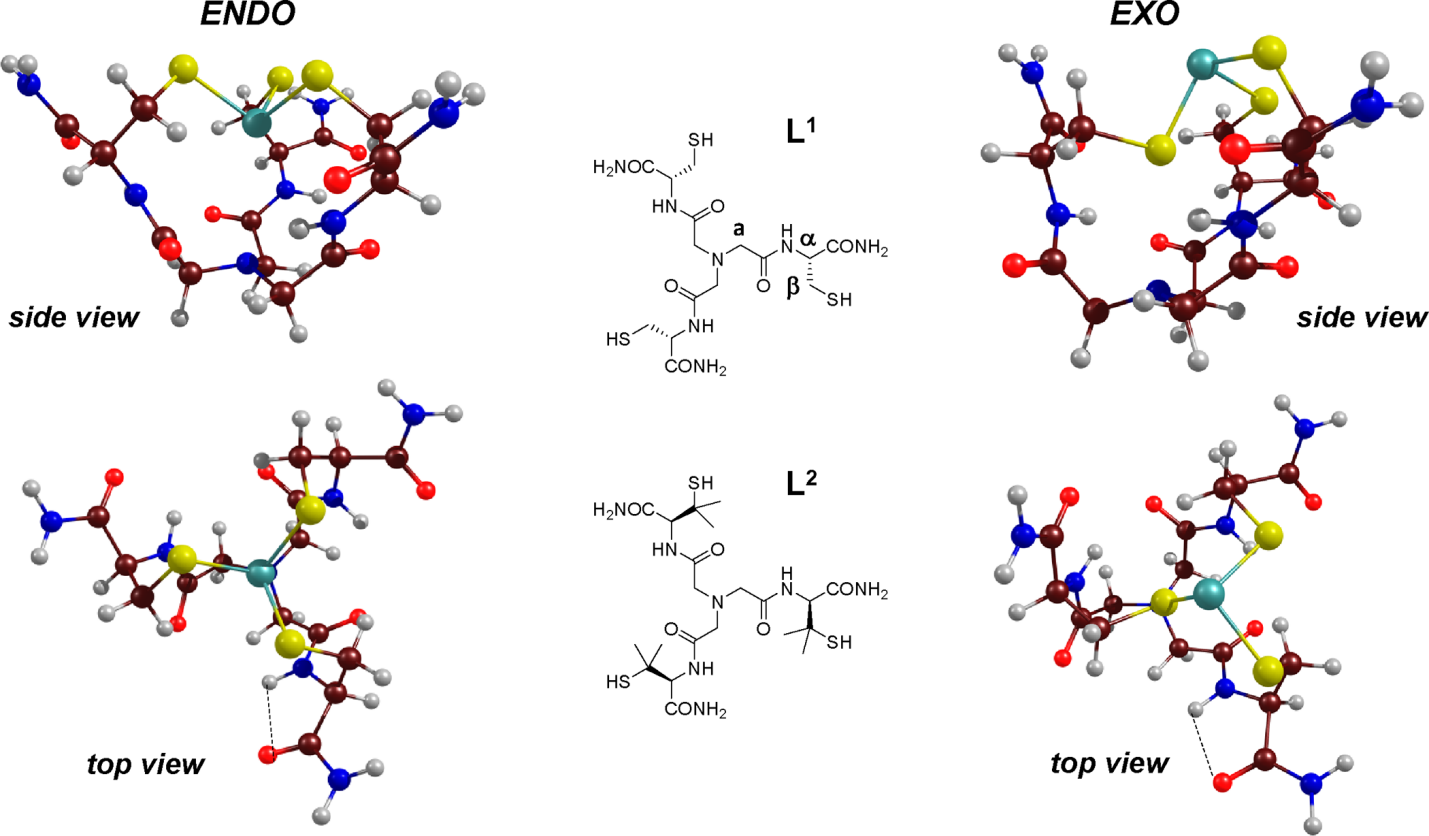
Schematic Structure of the Ligands NTA(Cys-NH_2_)_3_ (**L^1^**) and NTA(d-Pen-NH_2_)_3_ (**L^2^)** (Middle) with Notations of the Hydrogens of **L^1^** Monitored by ^1^H NMR Spectroscopy and the B3LYP-D3/def2-tzvp Optimized Structures of the Energetically Favored *endo,endo,endo* Conformer (Left) and the Less Favored *endo,endo,exo* Conformer (Right) of the As**L^1^** Species in Side and Top Views Color code: arsenic, turquoise; sulfur, yellow; oxygen, red; nitrogen, blue; carbon, brown; hydrogen, white.

In this paper, we show that simple bioinspired pseudopeptides, offering a tristhiolato coordination site, induce an AsS_3_ coordination only. The two ligands **L^1^** (NTA(Cys-NH_2_)_3_) and **L^2^** (NTA(d-Pen-NH_2_)_3_), shown in Scheme 1, were chosen to mimic the AsS_3_ coordination found in some biological As^III^ binding sites, thanks to a nitrilotriacetic acid anchor grafted with either three cysteine or d-penicillamine moieties. The two ligands display three thiol functions in a tripodal architecture and are prone to offer a tristhiolato coordination site to soft metal ions. A C-terminal neutral primary amide instead of an acid was preferred for the three amino acids to avoid interferences for metal coordination and also electrostatic repulsions in the final complexes at physiological pH. These two sulfur ligands were previously demonstrated to promote a MS_3_ coordination with Hg^II^ and Cu^I^ in aqueous solution buffered at pH 7.4.^16−19^ Therefore, we anticipated that **L^1^** and **L^2^** could be of interest to model As^III^ binding in an AsS_3_ coordination. By contrast to Hg^II^ and Cu^I^, which are soft metal cations with a 5d^10^ and 3d^10^ electronic configuration, respectively, As^III^ is a metalloid having a 3d^10^4s^2^ electronic configuration with the presence of a lone pair that may strongly influence its coordination, as described above. In this work, a detailed analysis of the intimate As^III^ coordination sphere in the two As^III^**L^1^** and As^III^**L^2^** biomimetic complexes was performed with various experimental data, which were confronted to density functional theory (DFT) calculation. This revealed a unique AsS_3_ binding site with a structure reminiscent of sites found in As^III^-binding proteins.

## Experimental Section

### Materials

The studied ligands were synthesized and purified, as described before.^16,17^ All other chemicals, purchased from Sigma-Aldrich (Merck), were of analytical grade and used without further purification. (Caution: The solid compound arsenic(III) oxide is a chemical with high health hazards (acute toxicity, skin corrosion, serious eye damage, and carcinogenicity) and environmental hazards (long-term (chronic) aquatic hazard) and should be handled with extreme care using the necessary protective equipment. Any solution samples containing the aqueous forms of arsenic(III) are also toxic and must be handled/disposed according to the regulations for toxic liquid wastes.) Aqueous solutions were prepared by using Milli-Q water of ultrapure laboratory grade.

### Preparation of Stock Solutions and Samples for Spectroscopic Studies

Stock solutions of the ligands were prepared in an oxygen-free argon atmosphere in a glovebox every second day or before a new series of experiments. The solid compounds were dissolved in the buffer solution/solvent used for the specific experiment. The medium of a typical sample was phosphate buffer (20 mM, pH = 7.0). The ionic strength of the samples was adjusted to 0.1 M by NaCl. The concentrations of the stock solutions of the ligands varied between 5.0 × 10^–5^ and 1.3 × 10^–4^ M and were determined by Ellman’s procedure. According to the procedure, 5,5′-dithiobis(2-nitrobenzoic acid) (DTNB) reacts with the free thiol groups of the ligands and produces a 2-nitro-5-thiobenzoate anion (TNB^2–^) per each reacting thiol group that is detected at 412 nm (ε = 14 150 M^–1^ cm^–1^).^20,21^ Stock solutions of arsenous acid were prepared weakly by dissolving a precisely weighed mass of arsenic(III) oxide in a 0.1 M NaOH solution (ca. 2–2.5 mg of As_2_O_3_ per 1.0 mL) with heavy stirring under an argon atmosphere (glovebox). The alkaline solution was neutralized by a 1.0 M HCl solution to a pH value between pH ∼4 and 6. The final concentration of arsenous acid was adjusted to 1.0 × 10^–2^ M by adding the necessary volume of Milli-Q water. Samples were prepared by mixing stock solutions of the ligands and arsenous acid.

### Recording UV–Vis Absorption and Circular Dichroism (CD) Spectra

The UV–vis absorption spectra were measured by using optical fibers connecting an external cell holder, placed in the glovebox, with a Varian Cary 50 spectrophotometer (operating outside the glovebox). The spectra were recorded in a quartz cuvette of a 1.0 cm path length in the wavelength range of 220–550 nm. The baseline was recorded for the empty cell holder and the spectrum of the cuvette, filled with the buffer/solvent used for the preparation of the relevant samples, was subtracted from the spectra of the samples. Data from the wavelength range of 240–335 nm was used for calculating stability constants for As^III^ binding by **L^1^** and **L^2^** (vide infra). The typical concentrations of the ligands were 4.1–5.0 × 10^–5^ M (**L^1^**) and 1.0 × 10^–4^ M (**L^2^**). Aliquots of arsenous acid stock solutions (or base in pH titrations) were gradually added to the ligand solutions and the spectra were acquired after a ca. 10 min waiting time allowing for equilibration. The CD spectra were measured with an Applied Photophysics Chirascan photometer. The instrument was calibrated by using (1*S*)-(+)-10-camphorsulfonic acid as a reference compound. Data were collected in the wavelength range of 350–190 nm with 1 nm bandwidth and 2 s dwell time per data point. Three scans were measured for each sample, including the background buffer solution. The average spectrum of the scans was corrected by the spectrum of the background and smoothed by the Savitzky–Golay method with a “window size” of 7. Each sample and each addition of the arsenous acid solution of a titration series was performed in the glovebox under an argon atmosphere in a quartz cuvette, equipped with a Teflon cap. The CD instrument was operated outside the glovebox. The titration protocols were similar to those applied for the UV–vis studies. The concentrations of the ligands were 2.5 × 10^–5^ M (**L^1^**) and 1.0 × 10^–4^ M (**L^2^**).

### Recording ESI-MS Spectra

ESI-MS experiments were performed on a Thermo Scientific LXQ-linear ion trap instrument equipped with an electrospray ion source. The full scan spectra were acquired in the range of *m*/*z* = 50–1500 or 50–2000 amu in the positive ion polarity mode (**L^2^** samples) and in the positive and negative ion polarity modes (**L^1^** samples) by infusion through a fused silica tubing at a flow rate of 2–10 mL min^–1^. The instrument calibration (*m*/*z* = 50–1500) was achieved by following the standard calibration procedure from Thermo Scientific (using a mixture of caffeine, MRFA, and Ultramark 1621). The heated capillary for the LXQ was set in the temperature range of 200–250 °C, while the ion-spray voltage was adjusted in the range of 3–6 kV. The injection time varied between 5 and 200 ms. Samples of **L^1^** and **L^2^** were prepared in ammonium acetate buffer (20 mM, pH = 6.9) with ligand concentrations of 100 μM. Aliquots of arsenous acid solutions were added to set the desired As^III^:**L^1^** (or **L^2^**) concentration ratio.

### ^1^H NMR Experiments

NMR measurements were carried out on a Bruker Avance 400 MHz spectrometer operated at 400.13 MHz (for ^1^H). The 1D ^1^H NMR spectra were recorded with a spectral width of 10 ppm and a 32,000 time domain by using 64 scans. Samples were prepared in a phosphate buffer in D_2_O (*c*_buffer_ = 20.0 mM, pD = 7.21) using **L^1^** in a concentration of *c* = 90–100 μM. As^III^ was added to the samples of **L^1^** from a stock solution of arsenous acid prepared in D_2_O by using NaOD for the dissolution of As_2_O_3_ and DCl for pD adjustment. To gain a clearer insight on the coordination mode of the As^III^-bound **L^1^**, a ^1^H–^1^H gCOSY experiment, using the *cosygpppqf* pulse program, was also carried out in an As^III^:**L^1^** 1:1 sample. The 2D spectrum was acquired with 2048(F2) and 256(F1) complex points each in a spectral width of 3.5 ppm applying 280 scans. All spectra were processed by the Topspin 4.0.7 software (Bruker).

### DFT Calculations and Computational Details of Simulating UV and CD Spectra and ^1^H NMR Chemical Shifts

DFT calculations were run with Orca 5.0.^22−24^ Geometry optimizations were carried out with the B3LYP hybrid functional including dispersion terms through the D3BJ correction. We checked that numerical frequencies were all real for the optimized geometries presented here. The solvent was taken into account using a polarizable continuum model (cpcm) for water. The all-electron def2-tzvp basis set was used for all atoms. Default integration and grid accuracy parameters were used, together with a TightSCF criterion. Starting geometries were built with a C3 geometry first with *endo,endo,endo* geometries and both possible helices (clockwise and anticlockwise). Optimization slightly distorted the trigonal symmetry for these all-*endo* structures as revealed by bond lengths, even with tight convergence criteria. From the C3 starting geometry, the *exo* ones were generated by moving the As atom outside the cavity along the C3 axis to generate an *exo,exo,exo* position. But the latter relaxed always to an *exo,endo,endo* conformation; thus, it loses the trigonal symmetry. Simulations of the UV and CD spectra were based on TDDFT approaches with 30 excitations in a continuum model for solvation in water, with B3LYP and def2-tzvp basis sets. ^1^H NMR chemical shifts were obtained by calculating the shielding tensor within the gauge-including atomic orbital (GIAO) origin and using the TMS proton shielding tensor as the reference. The TPSS functional with pcSeg-2 basis set was chosen based on previous works.^25^ Building and displaying structures and spectra were done using Chemcraft.^26^ Electrostatic potential visualization was done with the AMS GUI package^27^ on single points with ADF 2016 (B3LYP and TZ2P basis sets) using the previously optimized geometries.

### Calculation of the As^III^ Binding Affinities of the Ligands

The spectra of the As^III^ titration series of **L^1^** and **L^2^** were evaluated by the PSEQUAD software^28^ using absorbance values from the wavelength range of 240–335 nm. The fitting procedure provided conditional stability constant (log*K*′) for the AsL species, formed via the As + L ⇌ AsL equilibrium process at pH = 7.0, characterized by the equation *K*′ = [AsL]/([As] × [L]). In these formulae, As stands for the free arsenous acid, while L represents the non-coordinated ligand (**L^1^** or **L^2^**) irrespectively of their protonation states. Similarly, AsL is a general notion of the mono-complex adduct irrespectively of the form of the bound components. The molar absorption spectrum of the AsL mono-complex was also an output of the data evaluation process, while the molar absorption spectra of the free ligands at pH = 7.0 were calculated from the first spectra of the As^III^ titration series and used as a fixed set of parameters in the fitting procedure.

## Results and Discussion

Arsenic(III) binding by NTA-based tripodal scaffolds, displaying three thiol groups from cysteine (NTA(Cys-NH_2_)_3_ (**L^1^**)) or d-penicillamine (NTA(d-Pen-NH_2_)_3_ (**L^2^**)) arms (Scheme 1), was studied by a combination of UV–vis, CD, ^1^H NMR, and ESI-MS techniques, as well as by geometry optimization using DFT calculations.

### UV–Vis and CD Spectroscopic Studies

**Figure 1:**
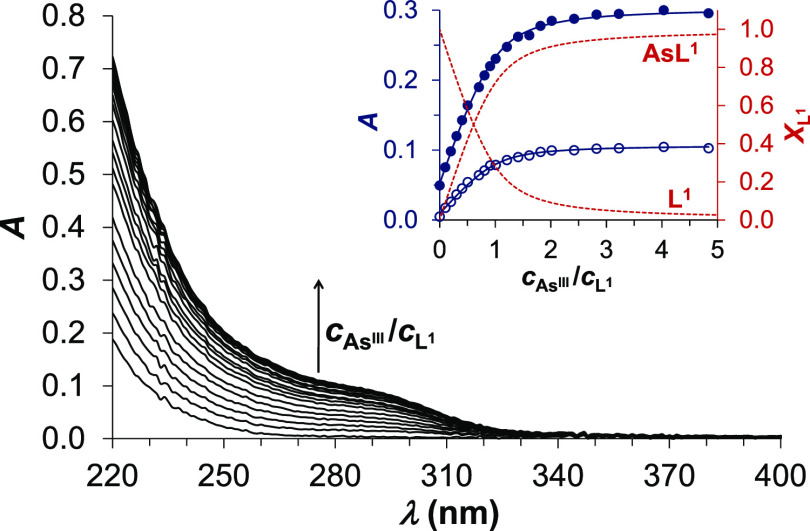
UV spectra of **L^1^** recorded in a titration with arsenous acid (As^III^) at pH = 7.0 (*c*_**L^1^**_ = 50 μM in phosphate buffer, 20 mM, *I* = 0.1 M NaCl). The inset shows the measured (shaded circle, 240 nm; unshaded circle, 280 nm) and fitted (continuous blue lines, left axis) absorbances as well as the relative fractions of the free and bound ligand (red dashed lines, right axis) as a function of the *c*_As^III^_/*c*_**L^1^**_ concentration ratio.

UV–vis-monitored titrations of **L^1^** at pH = 7.0 by solutions of arsenous acid reflect a monotonic absorbance increase between 220 and 350 nm, as a function of the As^III^:**L^1^** concentration ratio, leveling off only at a few-fold As^III^ excess over the ligand (Figure 1). The recorded spectra display a shoulder-like feature between 270 and 300 nm, very similar to that observed in the UV–vis spectra of As^III^-glutathione (GSH)^29,30^ and, with a less characteristic shoulder-shape, in the systems of dihydrolipoic acid (DHLA),^30^ dimercaptosuccinic acid (DMSA),^30^ or 2,3-dimercaptopropan-1-ol (BAL)^31^ under conditions where the coordination of three sulfur donors to As^III^ has been suggested. The same type of band, assigned to S^–^ → As^III^ ligand-to-metal charge transfer transitions (LMCT),^29,30,32−34^ seems to occur at slightly higher energies (250–270 nm) when only two thiolates are bound to the As^III^ center, as exampled by data for dithioerythritol (DTE)^32^ and dithiothreitol (DTT).^30^ The molar absorption coefficient calculated for the proposed As**L^1^** complex (see below) is ε_280nm_ = 2.2 × 10^3^ M^–1^ cm^–1^, which is rather similar to what was reported for the As(GSH)_3_ complex,^29^ and significantly larger than the estimated values in species with two As^III^–thiolate bonds (ε_280nm_ ∼4.8–6.4 × 10^2^ M^–1^ cm^–1^; ε_260–270nm_ ∼0.85–1.0 × 10^3^ M^–1^ cm^–1^).^30−32^ The position and intensity of the LMCT band for As^III^-**L^1^** as well as the lack of notable spectral changes between pH ∼6.5 and 8.4 (see Figure S1) all point to a tristhiolate coordination of **L^1^** to As^III^. A pH-dependent series of spectra, recorded for the sample containing As^III^ and **L^1^** in a 2:1 ratio, suggests a gradual decomposition of the As**L^1^** mono-complex above pH ∼8.4 (Figure S1). This process liberates the deprotonated free ligand, leading to the emergence of the *n* → σ* transition of the thiolate moieties, centered near 240 nm^29,31,32^ (Figures S1 and S2). This is similar to what was observed with BAL^31^ or DTE,^32^ although only at higher pH (above pH ∼10.0), which might be a consequence of the higher affinity of As^III^ to the latter dithiolate molecules, which exhibit a strong chelate effect due to the formation of a five-membered ring involving the two As-bound thiolates, deferring the hydrolysis of the As^III^–thiolate bonds (see below).

**Figure 2:**
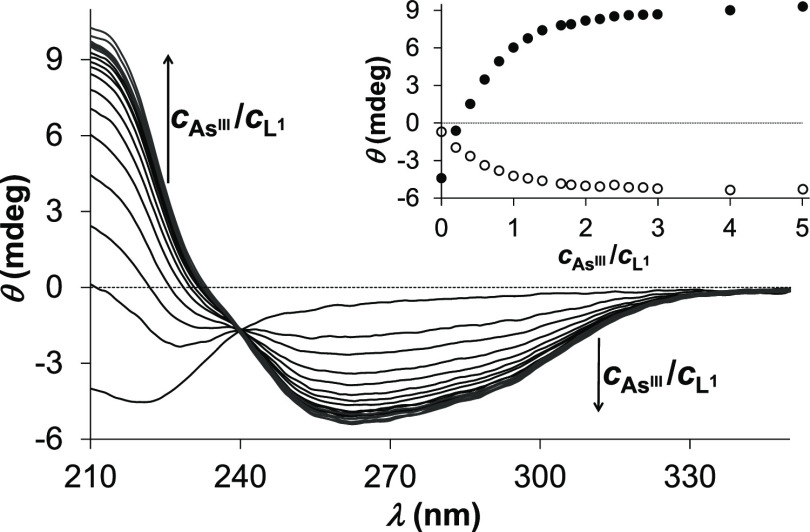
CD spectra of **L^1^** recorded in a titration with arsenous acid at pH = 7.0 (*c*_**L^1^**_ = 25 μM in phosphate buffer, 20 mM, *I* = 0.1 M NaCl). The inset shows the measured ellipticity traces at λ = 216 nm (shaded circle) and at λ = 260 nm (unshaded circle).

Gradual addition of arsenous acid to the solution of **L^1^** shows the same saturation-like tendency in the recorded CD spectra as in the UV–vis data, i.e., the complexation process is completed only at a few-fold As^III^ excess over **L^1^** (Figure 2). A single isodichroic point around 240 nm, characteristic for the whole monitored *c*_As^III^_:*c*_**L^1^**_ concentration ratio range, indicates that only one **L^1^** complex is in equilibrium with the free ligand. CD spectroscopic data on systems of “colorless” metal ions and ligands with chirality centers near the metal ion binding groups^16,17,19,35−37^ indicate that the positive and negative CD bands with intensity extrema near 210 and 260 nm, respectively, can be assigned to the LMCT transitions (vide supra). The optical activity of **L^1^** is efficiently propagated from the α carbon chirality centers toward As^III^ via the As^III^–thiolate bonds. Similar to this, LMCT-related CD features also emerged in the UV region accompanying the coordination of the thiolate groups of **L^1^**(16) and **L^2^**(17 ,19) to Hg^II^ and Cu^I^.

UV–vis and CD titrations performed for samples of As^III^-**L^2^** (Figures S3 and S4) show similar trends to those for As^III^-**L^1^**, characterizing a simple equilibrium between the free **L^2^** and a tristhiolate-coordinated complex As**L^2^**. However, the obtained absorbance and CD intensity vs As^III^:**L^2^** concentration ratio profiles indicate a weaker As^III^ binding affinity of this bulkier compound that leads to a significant fraction of the ligand remaining unbound under the applied conditions even at high As^III^ excess.

### Results of ESI-MS Experiments

**Figure 3:**
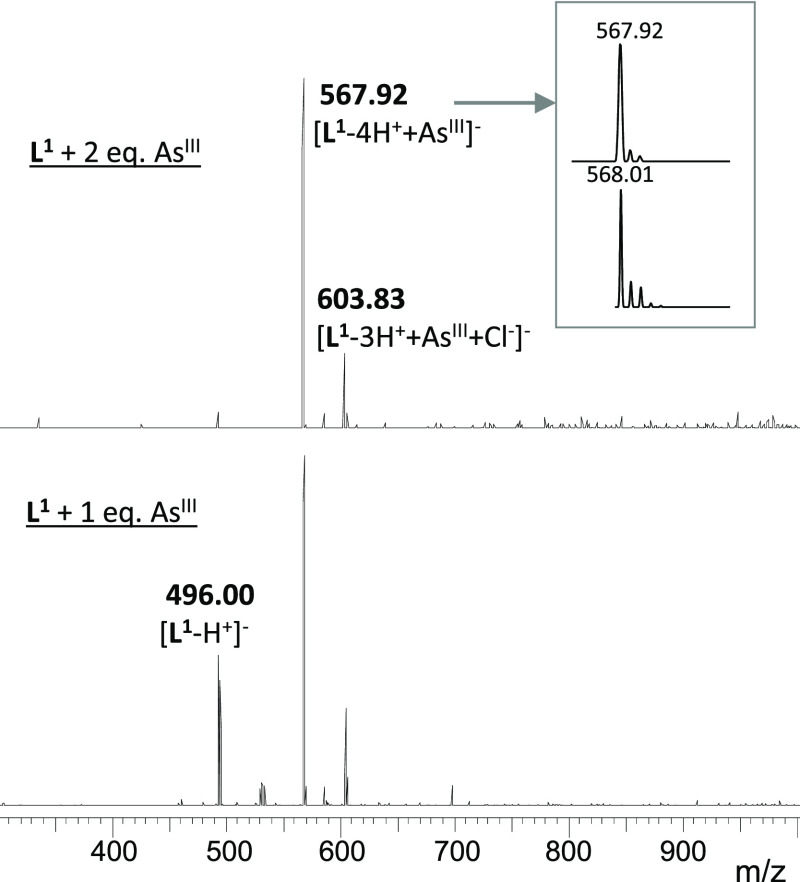
(−)ESI-MS spectra obtained for **L^1^** with 1 eq. (down) and 2 eq. (top) of As^III^ at pH = 6.9 (ammonium acetate buffer 20 mM).

ESI-MS studies confirm the presence of one major complex species of a 1:1 As^III^:**L^1^** ratio, which displays isotopic patterns corresponding to [**L^1^** – 4H^+^ + As^III^]^−^ and [**L^1^** – 3H^+^ + As^III^ + Cl^–^]^−^ compositions in the negative ion mode (Figure 3), which supports the binding of all the three thiolates to As^III^ in a monomeric species. ESI-MS reflects the presence of monomeric species of **L^2^**, too; however, signals of these adducts are very weak compared to those of the free ligand. Even in excess of As^III^, for instance, for a 2:1 As^III^:**L** ratio (Figure S5), where the spectrum acquired with **L^1^** shows only the As**L^1^** complex (Figure 3 top), the As**L^2^** species is barely detected. This qualitatively implies that the binding of **L^2^** to As^III^ is less efficient, which correlates well with the results of UV–vis- and CD-monitored titrations.

### Analysis of NMR Data

**Figure 4:**
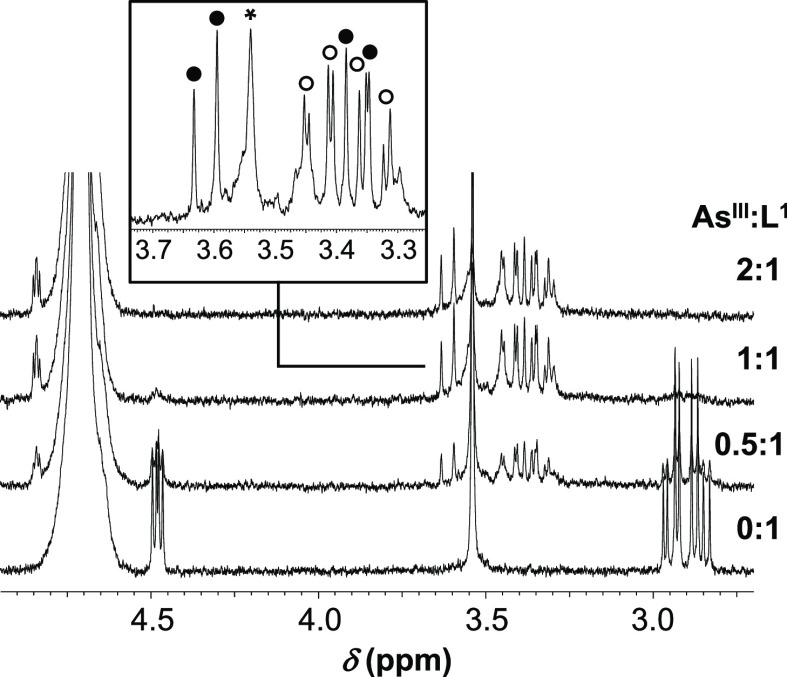
Selected region of the ^1^H NMR spectra of **L^1^** in a titration by arsenous acid (*c*_**L1**_ = 100 μM in D_2_O, phosphate buffer, 20 mM, pD = 7.21). The framed section of a separately recorded spectrum displays resonances of the Cys CH_2_β (unshaded circle) and the NTA-CH_2_ apical hydrogen atoms (shaded circle) of the bound ligand; * denotes residual resonances of the latter hydrogen atoms in the unbound ligand.

^1^H NMR data were also acquired for As^III^-**L^1^** to gain insight into the structure of the tristhiolate-bound species. Resonances of the free ligand gradually diminish, while new signals for the bound ligand, with significantly larger chemical shifts for the CHα and CH_2_β and altered splitting pattern of the NTA-CH_2_ apical hydrogens (Figure 4 and Figure S6), emerge in parallel with the increasing As^III^:**L^1^** concentration ratio (Figure 4). This indicates a slow ligand exchange at the NMR timescale in the As**L^1^** complex, which is in accordance with previous findings indicating a limited lability of the ligands bound in As^III^ complexes via two or three As–S bonds.^30,31,38^ The splitting of the singlet of the NTA-CH_2_ apical hydrogens of the unbound ligand into an AB spin system in As**L^1^** is a consequence of the blocked motion of the As-anchored ligand arms. In addition, one major resonance pattern is observed for the three NTA-CH_2_, the three CHα, and for the three CH_2_β groups reflecting a C_3_ symmetry of the complex. A couple of other low-intensity signals (e.g., at ca. 3.30 and 3.45 ppm; Figure 4 and Figure S6) may, however, indicate the presence of minor isomeric structures, too. These NMR data well support the findings of all other experiments and point to one major tristhiolate-coordinated species displaying a highly symmetric orientation of the tripod arms around the semimetal center.

### Determination of the Stability of Species Formed under Neutral Conditions

The binding affinity of **L^1^** and **L^2^** to arsenous acid was quantitatively assessed by evaluating UV–vis data (Figure 1 and Figure S3) in the wavelength range of 240–335 nm by using the PSEQUAD PC software.^28^ The ligands are bound to arsenous acid in condensation reactions with no pH effect, as one water molecule per each forming As–S bond is released.^31,32^ Accordingly, apparent stability constants for pH = 7.0 (log*K*′), characterizing the simple As + **L** ⇌ As**L** equilibrium process with As denoting the free arsenous acid and **L** (**L^1^** or **L^2^**) the unbound ligand in any protonation state, were calculated, as described previously.^31^ The measured and calculated absorbance traces in Figure 1 (and in Figure S3), together with the relative fractions of the bound and unbound ligand, demonstrate that this simple model adequately describes the complexation processes: log*K*′_As**L^1^**_ = 5.26 ± 0.02 and log*K*′_As**L^2^**_ = 3.04 ± 0.01, converted into *K*_d,As**L^1^**_ = 5.5 μM and *K*_d,As**L^2^**_ = 910 μM dissociation constants. Taking into account the similar basicities of the cysteine and penicillamine sidechain thiol groups,^17^ the two orders of magnitude difference in the obtained stability values indicates a strong destabilizing effect of the methyl substituents in the d-Pen derivative.

### Computational Studies

DFT molecular modeling was used to correlate the experimental data (especially those from NMR and the relative stabilities of the As**L^1^** and As**L^2^** species) with the most probable As^III^-bound ligand structures. Previous theoretical (DFT) studies on various trigonal pyramidal As^III^ complexes showed that the conformation of the three As^III^-bound moieties is not necessarily identical (*endo* or *exo*) in the energetically most favored structures.^7,34^ Therefore, DFT geometry optimizations were launched from C_3_ symmetric geometries of **AsL^1^** and **AsL^2^** with both possible helical configurations (named below hel1 and hel2) either from an all-*endo* (*endo,endo,endo*) or from an all-*exo* (*exo,exo,exo*) structure. The former yielded the most stable all-*endo* structures, whereas the latter relaxed systematically to an asymmetric mixed *endo*,*endo*,*exo* orientation of the S-CH_2_ groups, higher in energy than the *endo* conformers (see Figures S7 and S8 and Tables S1 and S2). Scheme 1 displays the most stable all-*endo* and one *endo,endo,exo* conformers for **AsL^1^**. The calculated As–S bond lengths (2.24–2.29 Å; Tables S3 and S4) are similar to those reported for low-molecular-weight As^III^ complexes,^32,39^ the trigonal pyramidal AsS_3_ sites in three-helix bundles of oligopeptides,^40,41^ or As^III^-binding proteins.^3,5,6,42,43^ Some H-bonding features are notably present inside one peptide arm and between two arms of the trigonal structure (Tables S3 and S4).

To confirm the all-*endo* to be the most stable structure, we simulated the UV–vis absorption spectra (Figure S9) and CD spectra (Figure S10) for **AsL^1^** using time-dependent DFT approaches (see the Experimental Section). Whereas the absorption spectra do not allow to discriminate between conformers, the CD-simulated spectra are more significant. The *endo* hel1 CD spectrum clearly exhibits the same trend as the experimental one. Finally, some significant ^1^H chemical shifts were calculated for **AsL^1^** and are found to be similar to the experimental ones (Table S5). The same conformational ordering is obtained for the **AsL^2^** species with an all-*endo* conformer as the most stable one (Table S2).

**Figure 5:**
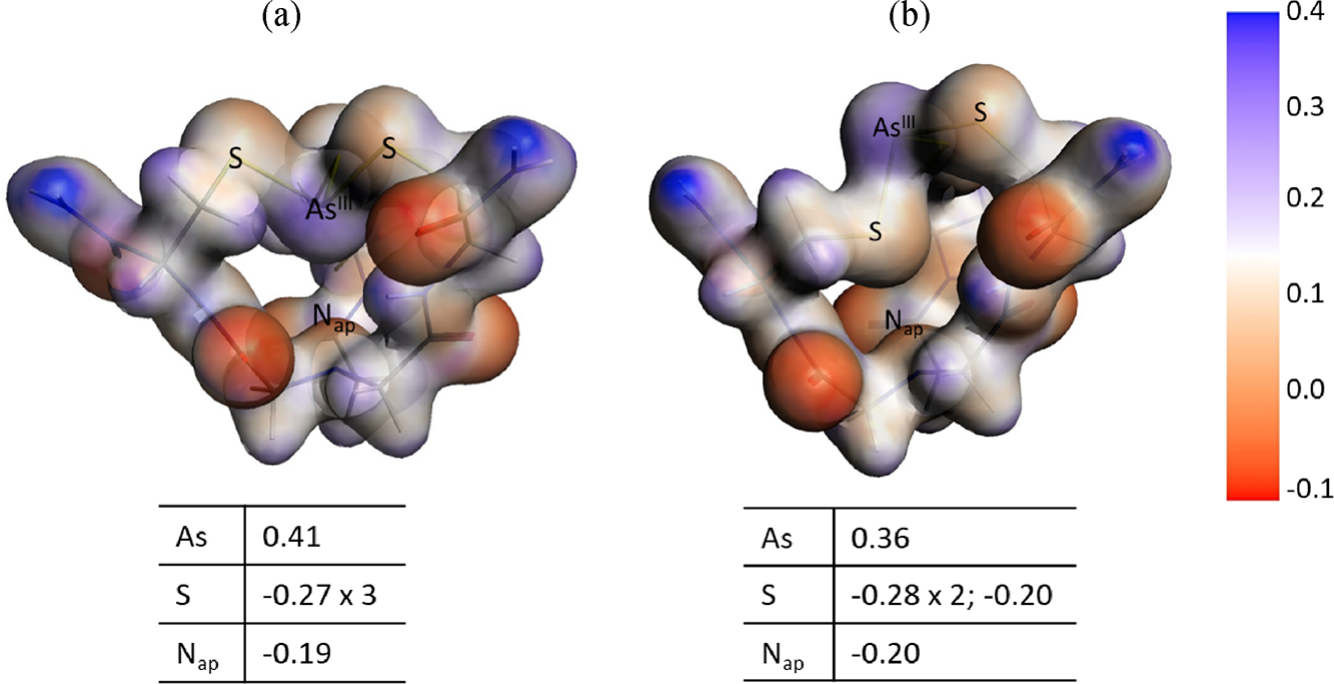
Electrostatic potential (in a.u.) projected on the electron density isosurface for **AsL^1^** together with Mulliken charges (As, S, and N_ap_ atoms) on hel1 conformers: (a) *endo* and (b) *exo*.

The reasons why the all-*endo* conformers are the most stable structures although the lone pairs of As^III^ and of the apical N atom point toward each other into the cavity may be found by careful examination of conformational as well as electronic properties of the As^III^ complexes. First, an isocontour plot of the electron density (Figure S11) shows that the lone pairs are not interacting. Additionally, previous DFT calculations on a small model As(SMe)_3_ revealed that the most stable configurations were in a narrow energy range (2.2 kcal/mol), and particularly the all-*endo* conformer was only 1 kcal/mol higher than the most stable *endo,endo,exo* one.^7^ This means that other environmental effects may control the final, most favorable configuration as it was demonstrated in the various protein models chosen in this study. In our case, the relative energy ordering of the ligands alone, taken in the geometry of each of the conformers (Tables S1 and S2), follows the same order as that of the As^III^ complexes, i.e., the all-*endo* ligand conformation is more stable than the *endo,endo,exo* one. Thus, the stabilization effects arising from the ligands themselves, such as intra- or inter-arm H-bonding, are not disrupted in the As^III^-bound species. Moreover, the actual interaction between As^III^ and the apical nitrogen atom in both all-*endo* and *endo,endo,exo* conformers was examined based on atomic Mulliken charges and the electrostatic potential surface (Figure 5). The all-*endo* conformer provides a favorable electrostatic interaction with opposite charges on As and N, whereas the sulfur lone pairs point outside the cavity. Numerous structural combined to computational studies have described the nature of the pnictogen bond between As^III^ trihalide species (mainly with F^–^ or Cl^–^) and electron donor species such as N-, Se-, S-containing molecules or hexaalkylbenzene rings.^13,44−47^ They all show donor–acceptor interactions, with As^III^ being the electrophilic agent. Thanks to simple models, bond orientation preferences due to the so-called σ- or π-holes along the As lone pair have been described.^13,44,46^ In a trigonal thiolate environment, the sulfur atom is expected to form a more covalent bond with As^III^, thus lowering its electrophilic character. Nevertheless, it has been shown by Vickaryous et al. that such a trigonal As(SR)_3_ moiety is able to provide some stabilization interaction with aromatic rings,^11^ as had been observed with As^III^ trihalides.^47,48^ The stabilization observed in the *endo,endo,endo* conformation of As**L^1^** is thus in line with these previous observations. By contrast, the *endo,endo,exo* conformer displays a sulfur atom inside the cavity, which thus exhibits repulsive electrostatic interactions with the apical nitrogen. The absence of all-*exo* structures may be explained by the inability in such structures to balance the steric effects of sulfur lone pairs with the required As–S bond lengths and the chelating organization of the tripodal ligand, as already noticed by Zampella et al.^7^ Indeed, in this study, the authors also pointed out that the most favored conformations of As(SMe)_3_ moieties are the *endo,endo,exo* and all-*endo* isomers differing only by 1 kcal/mol in energy. Interestingly, we observe that ligands **L^1^** and **L^2^** give energetically favorable conformations to accommodate the all-*endo* isomer. This observation evidences the good adjustment of the tripodal pseudopeptide ligands proposed in this study to stabilize the As^III^ coordination environment.

## Conclusions

In this work, we demonstrated that the NTA-based tripodal ligands bearing three Cys or d-Pen arms exclusively stabilize the AsS_3_ coordination environment of As^III^. The stability of the species formed with the sterically less restricted NTA(Cys-NH_2_)_3_ ligand is in the range determined (by a different method and under somewhat different conditions) for As^III^-oligopeptide complexes displaying two or three bound cysteines, with a slight possible extra stabilizing effect of the third coordinating sulfur.^49^ A combination of experimental and theoretical studies pointed to an all-*endo* conformer as the favored structure for the As^III^ complexes of both ligands, originating from stabilizing electrostatic interactions between the lone pair of As and the apical nitrogen atom.

The AsS_3_ coordination sphere imposed by the two biomimetic ligands is reminiscent of As^III^-bound protein or peptide species, with a preference for the all-*endo* conformer, which mimics the As^III^ coordination environment in the three cysteine-bound As^III^ semimetal sites in metalloproteins. Indeed, although the crystal structures found in the literature for the As^III^ complexes of bioligands, i.e., a three-stranded coiled-coil peptide,^50^ the metalloregulators CgArsR^6^ and AfArsR,^6^ or the ArsM protein,^5^ reflect some variability, all of these tristhiolate-coordinated semimetal centers display at least two *endo*-type S-CH_2_ carbon atoms around As^III^.

## Supporting Information Available

Tables of DFT-optimized parameters, figures of UV–vis, ESI-MS, and NMR spectra as well as of DFT-optimized structures (PDF)
