# Supplementary material for: Tristhiolato Pseudopeptides Bind Arsenic(III) in an AsS3 Coordination Environment Imitating Metalloid Binding Sites in Proteins
Source: Inorg Chem. 2023 Apr 18;62(17):6817–24. doi: 10.1021/acs.inorgchem.3c00563 (PMC10155180; doi:10.1021/acs.inorgchem.3c00563)
Supplement: Supplementary file 1 — ic3c00563_si_001.pdf [file ic3c00563_si_001.pdf]

Supporting Information

Tristhiolato Pseudopeptides Bind Arsenic(III) in a AsS_3 Coordination Environment Imitating Metalloid Binding Sites in Proteins

Levente Szekeres,[a†] Pascale Maldivi,[b] Colette Lebrun,[b] Christelle Gateau,[b] Edit Mesterházy,[a,b] Pascale Delangle[b] and Attila Jancsó[a]**

[a] Department of Inorganic and Analytical Chemistry, University of Szeged, Dóm tér 7, Szeged, H-6720, Hungary; [b] Universite Grenoble Alpes, CEA, CNRS, Grenoble INP, IRIG, SyMMES, Grenoble 38000, France

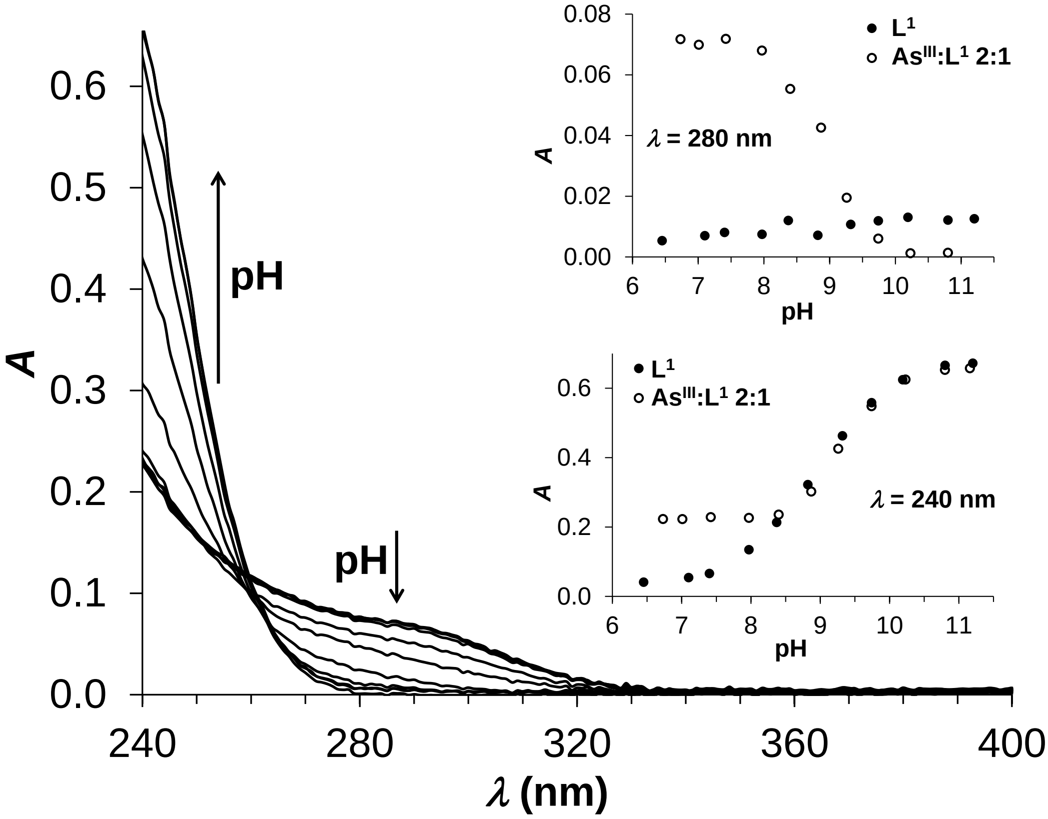

Figure S1. pH-dependent UV spectra recorded for the **L**^{1}-As^{III} 2:1 system (c_{L^1} = 41.7 μM, I = 0.1 M NaCl). Spectra are normalized for the dilution. The insets compare the trends in the change of absorbances measured in the presence (O) and absence (●) of As^{III} at 280 and 240 nm.

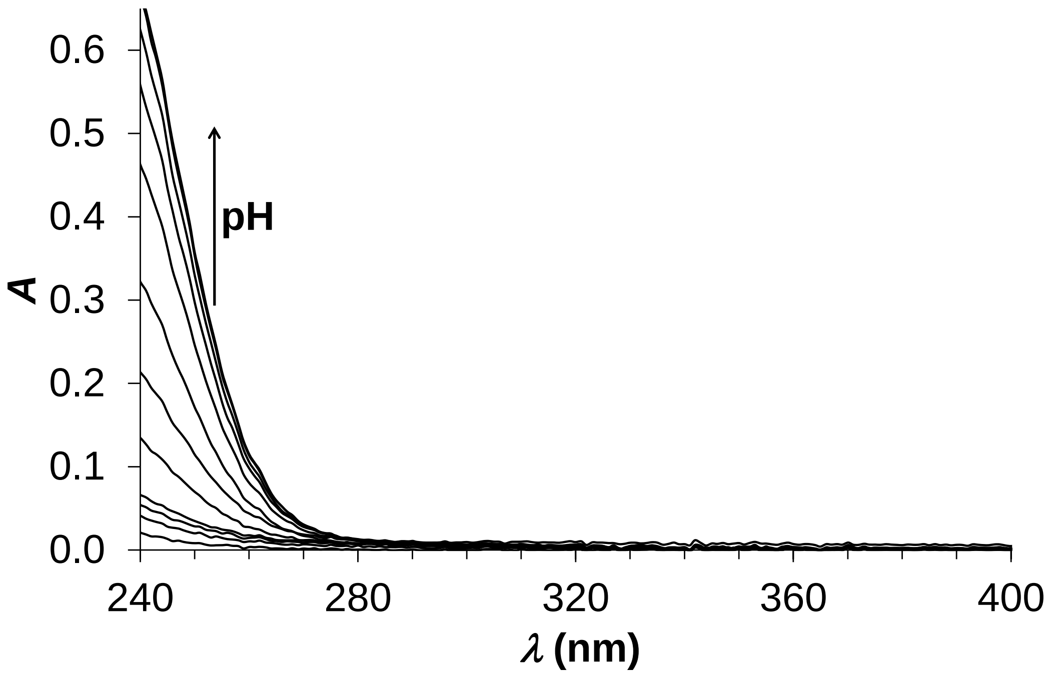

Figure S2. pH-dependent UV spectra of **L**^{1} (c_{L^1} = 41.9 μM, I = 0.1 M NaCl). Spectra are normalized for the dilution.

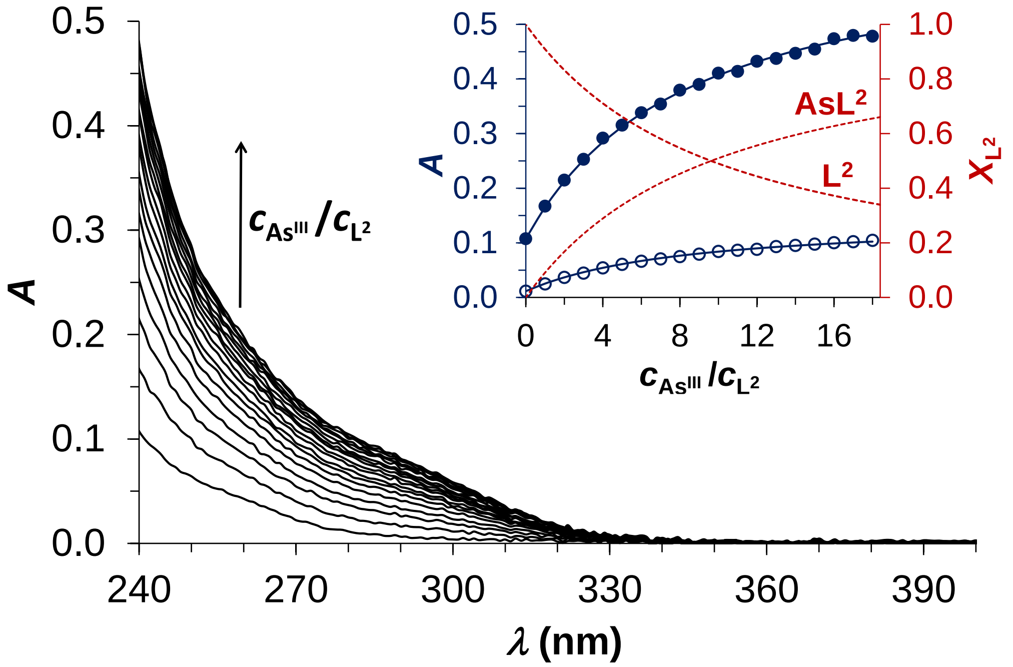

Figure S3. UV spectra of $\mathbf{L}^2$ recorded in a titration with arsenous acid (As^{III}) at pH = 7.0 (c_{L^2} = 100 μM in phosphate buffer, 20 mM, I = 0.1 M NaCl). The inset shows the measured (●: 240 nm, ○: 280 nm) and fitted (continuous blue lines - left axis) absorbances. While data of the titration shows similar trend to that of As^{III}-$\mathbf{L}^1$, indicating a simple equilibrium between the free $\mathbf{L}^2$ and a tristhiolate coordinated mono-complex As$\mathbf{L}^2$, a significant fraction of the ligand remains unbound under the applied conditions even at high As^{III}-excess (shown by the relative fractions of the free and bound ligand as red dashed lines in the inset - right axis), as a consequence of a weaker As^{III}-binding affinity of this bulkier compound.

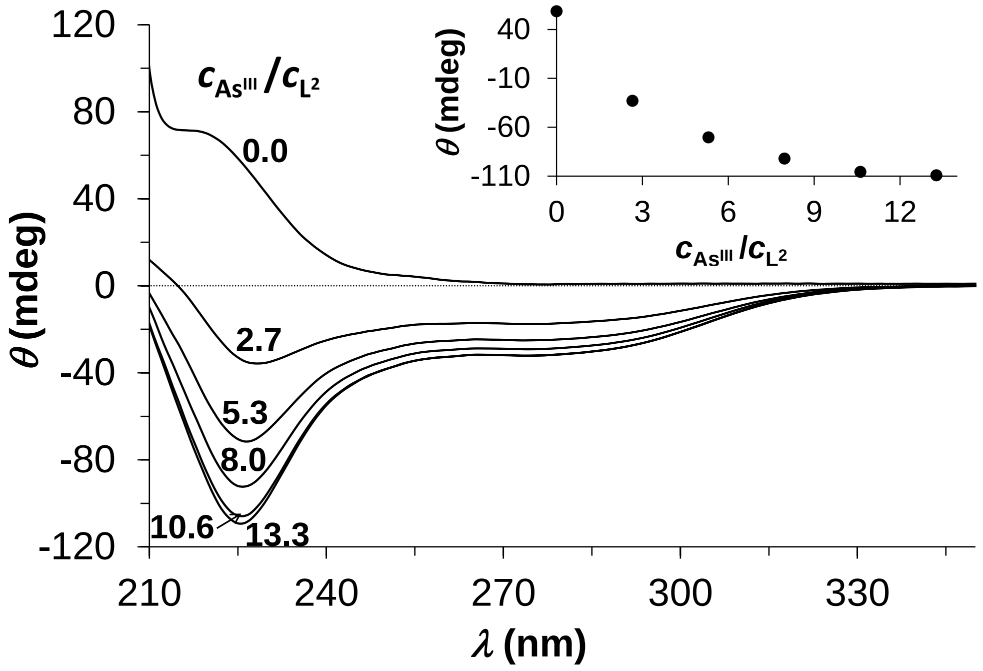

Figure S4. CD spectra of $\mathbf{L}^2$ recorded in a titration with arsenous acid at pH = 7.0 (c_{L^2} = 100 μM in phosphate buffer, 20 mM, I = 0.1 M NaCl). The inset shows the measured ellipticity traces at λ = 225 nm.

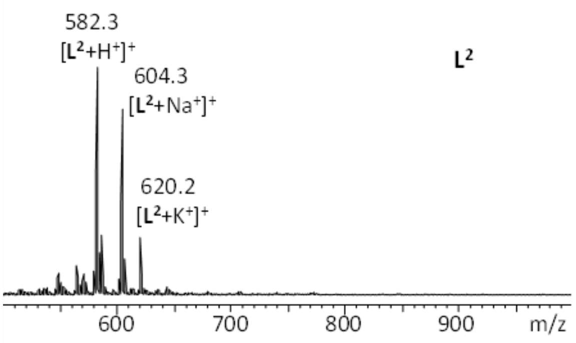

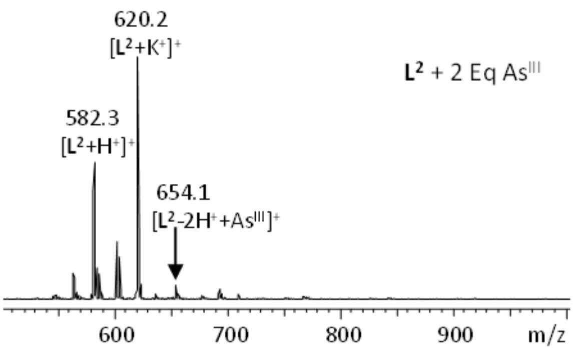

Figure S5. (+) ESI-MS spectra obtained for $\mathbf{L}^2$ in the absence (left) and presence of 2 eq. of As^{III} (right) at pH 6.9 (Ammonium acetate buffer 20 mM).

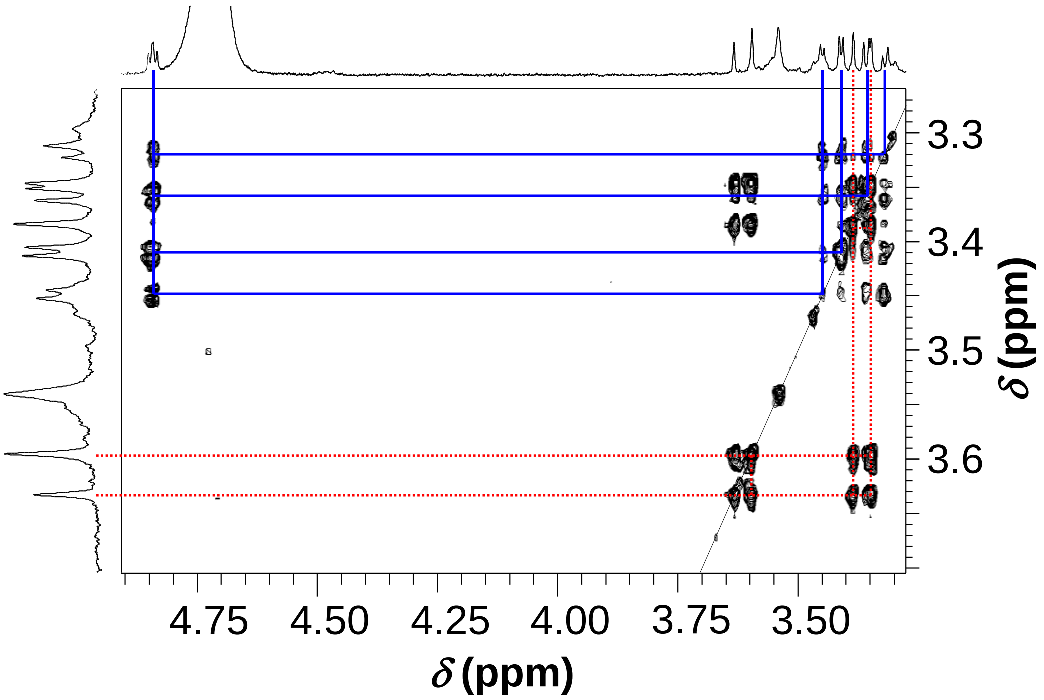

Figure S6. Part of the ^{1}H-^{1}H gCOSY spectrum recorded in the As^{III} : **L^1** 1:1 system at pD = 7.21 (c_{L^1} = 90 μM in D_2O - phosphate buffer, 20 mM, pD = 7.21). Continuous blue lines connect resonances of the CHα-CH_2β ABX spin system of the cysteine arms while the red dotted lines connect signals of the AB type spin system of the NTA-CH_2 apical hydrogens in the bound ligand.

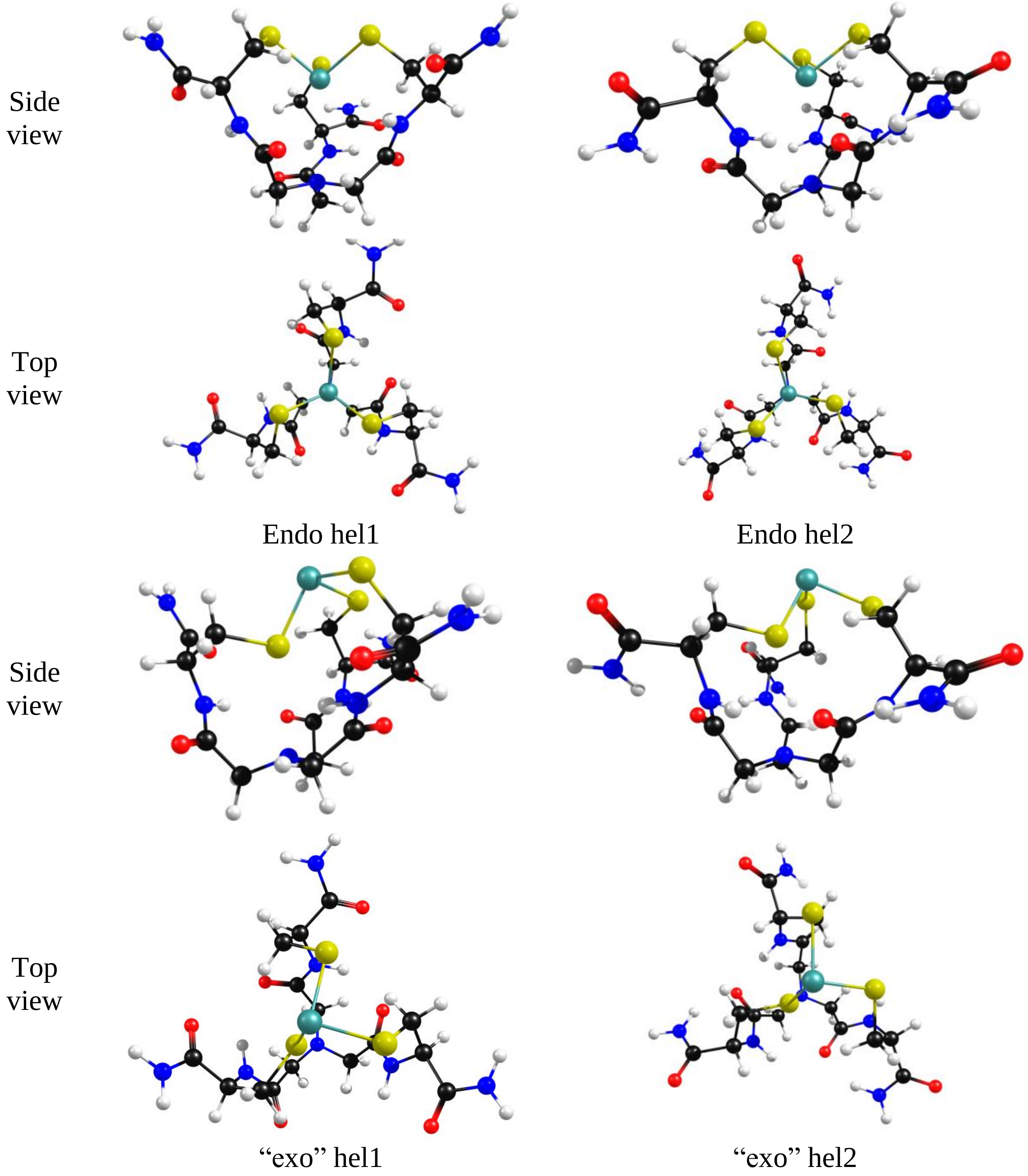

Figure S7. Optimized geometries of the four conformers of **AsL[1]** complex. The “exo” structure designates the *exo, endo, endo* conformer. Hel1 and hel2 correspond to clockwise and anticlockwise conformations of the ligand around the C3 axis (see top view). Color code: As turquoise, S yellow, oxygen red, nitrogen blue, carbon black, H white.

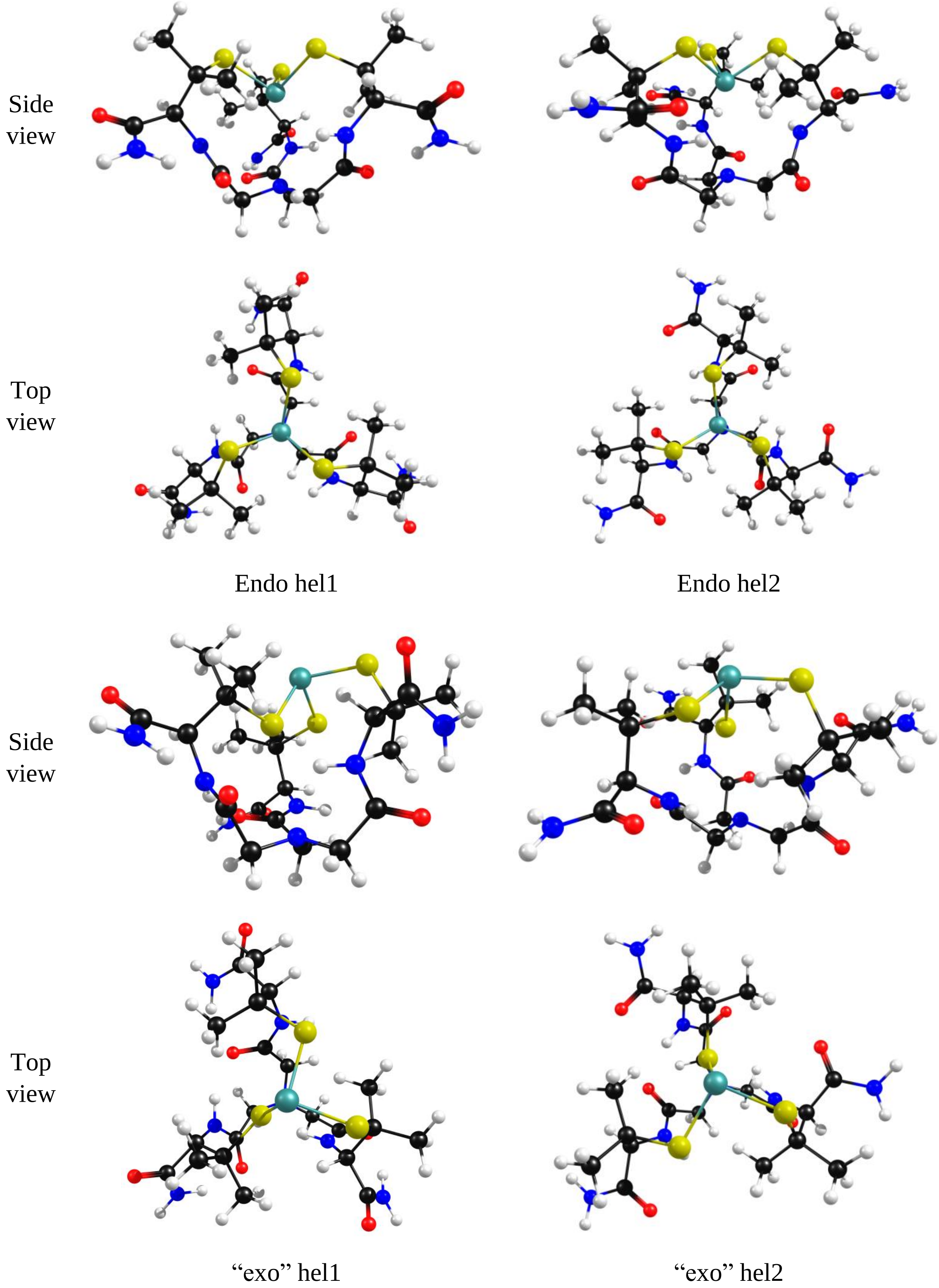

Figure S8. Optimized geometries of the four conformers of **AsL²**. The “exo” structure designates the *exo*, *endo*, *endo* conformer. Hel1 and hel2 correspond to clockwise and anticlockwise conformations of the ligand around the C3 axis (see top views). Color code: As turquoise, S yellow, oxygen red, nitrogen blue, carbon black, H white.

Table S1. Energetic ordering of the four conformations of **AsL^1** complex and of the **L^1** ligand alone, in the geometry of the complex (E in kcal/mol).

	ΔE (**AsL^1**)	ΔE (**L^1**)
endo hel1	0	0
endo hel2	6.1	5.4
exo hel2	9.5	15.5
exo hel1	14.6	16.2

Table S2. Energetic ordering of the four conformations of **AsL^2** complex and of the **L^2** ligand alone, in the geometry of the complex (E in kcal/mol).

	ΔE (**AsL^2**)	ΔE (**L^2**)
endo hel2	0.0	0
endo hel1	12.3	9.8
exo hel2	14.0	10.7
exo hel1	20.8	12.6

Table S3. Main bond lengths (Å) of the four conformations of **AsL^1** complex. See nomenclature in figure S1. H_N-O intra represents H-bond distance within one peptide arm (terminal carbonyl oxygen to NH amid group) and H_N-0 inter the H-bond distance between two branches.

	As-S	As-N(apical)	H_N-O intra	H_N-O inter
endo hel1	2.27	3.61	2.17 x 3	3.10; 3.20; 3.3
endo hel2	2.27	3.53	1.93 x 3	1.97; 2.65; 4.15
exo hel1	2.28 x 2; 2.24	5.37	2.17; 2.23; 2.35	2.16; 2.52; 3.15
exo hel2	2.29 x 2; 2.24	5.64	1.96 x 2; 1.86	2.00; 2.25; 4.30

Table S4. Main bond lengths (Å) of the four conformations of **AsL^2** complex. See nomenclature in figure S1. H_N-O intra represents H-bond distance within one peptide arm and H_N-0 inter the H-bond distance between two branches.

	As-S	As-N(apical)	H_N-O intra	H_N-O inter
endo hel1	2.28	3.43	1.84 x 3	3.30 x 3
endo hel2	2.28 x 3	3.49	2.31 x 3	3.7 x 3
exo hel1	2.26; 2.28 x 2	5.46	1.84 x 3	1.91; 3.17; 5.23
exo hel2	2.24 ; 2.29 x 2	5.64	2.34; 2.42; 2.75	2.04; 3.94; 5.13

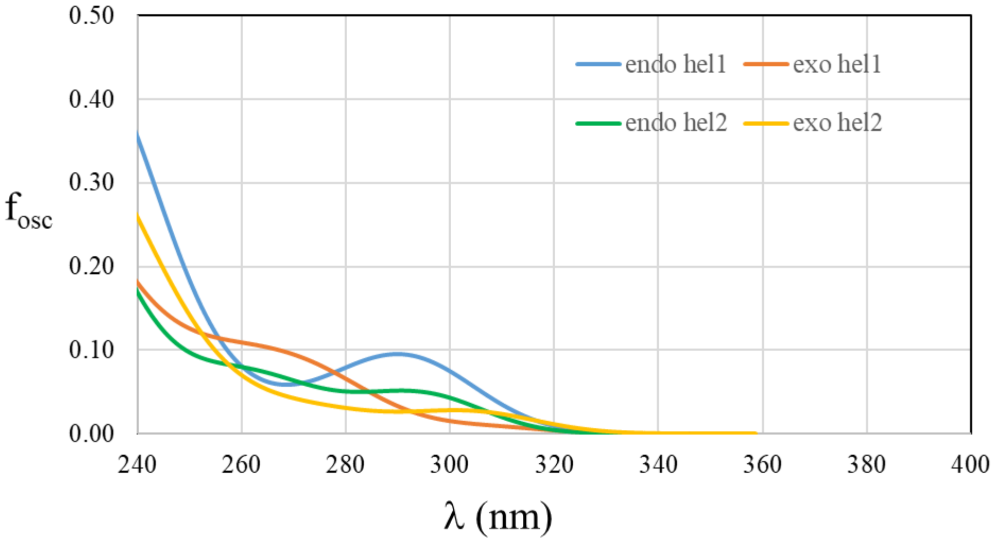

Figure S9. Computed UV spectra for the four conformers of **AsL**[1] represented as oscillator strength f_{osc} (arbitrary unit) vs wavelength (nm).

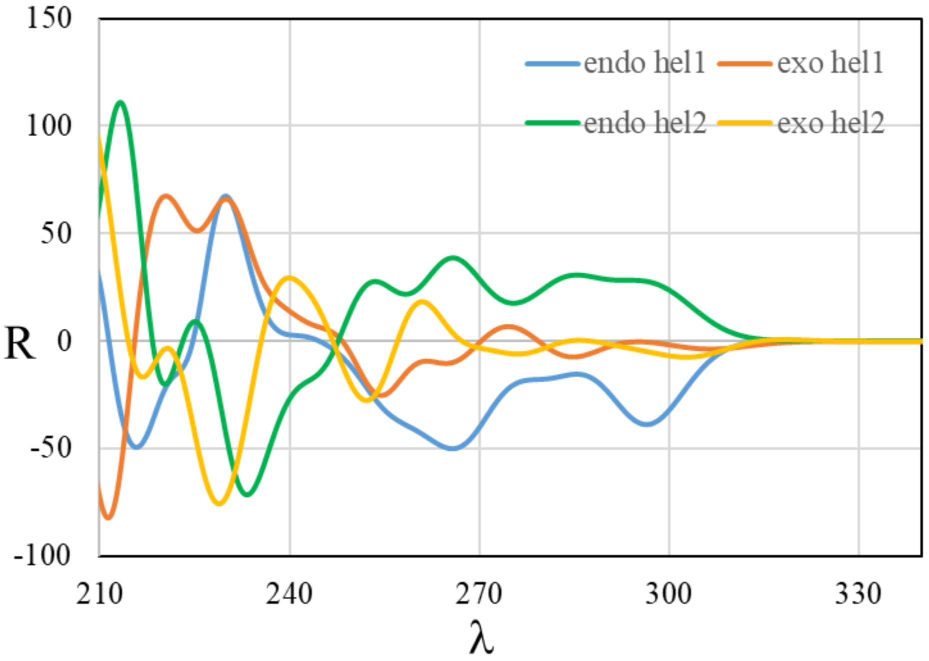

Figure S10. Computed CD spectra for the four conformers of **AsL**[1] represented as rotatory strength (10^{40} cgs) vs wavelength (nm).

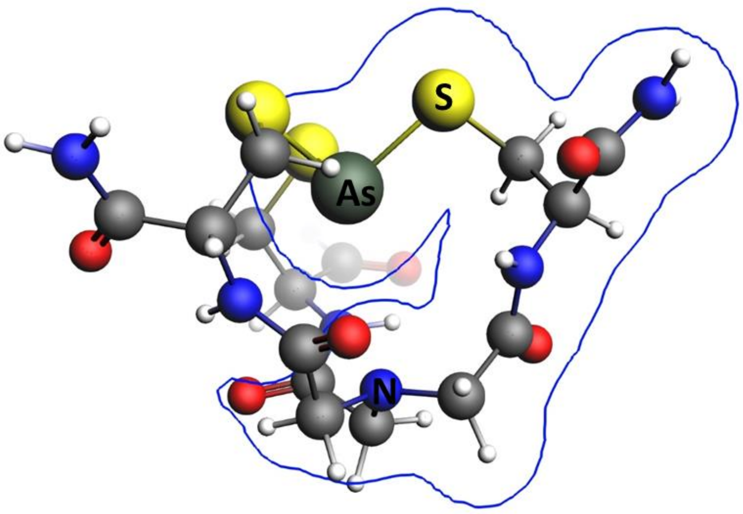

Figure S11. Electron density isocontour (0.004 ua) in a plane containing As, one S and the apical N atom.

Table S5. NMR chemical shifts of selected protons for **AsL[1]**.

	Exp. this work	DFT Hel1 C3 endo
**alpha**	4.84	4.7
**beta**	3.34	2.9
	3.43	3.3
**apical**	3 .61	3.6
	3.37	2.6
